# Combined Transcriptomic and Metabolomic Analyses of Low-Temperature Adaptation in *Bursaphelenchus xylophilus*

**DOI:** 10.3390/ijms27031470

**Published:** 2026-02-02

**Authors:** Xiong Xiong, Jie Li, Shuaibin Sun, Chengming Yu, Yehan Tian, Chuanrong Li, Huixiang Liu

**Affiliations:** 1 College of Forestry, Shandong Agricultural University, Tai’an 271018, China; 2019110362@sdau.edu.cn; 2 College of Plant Protection, Shandong Agricultural University, Tai’an 271018, China; jleesdau@163.com (J.L.); shuaibinsun@163.com (S.S.); ycm2006.apple@163.com (C.Y.); tianyehan@163.com (Y.T.)

**Keywords:** *Bursaphelenchus xylophilus*, low-temperature stress, transcriptomics, metabolomics, RNAi

## Abstract

*Bursaphelenchus xylophilus* (PWN), a highly destructive invasive forest pest, has expanded northward in China, even colonizing cold regions, implying evolved low-temperature tolerance. To explore its cold adaptation mechanisms, we selected PWN isolates from diverse origins, screened cold-tolerant strains via low-temperature stress assays, and conducted integrative transcriptomic and metabolomic analyses. We also compared invasive and native populations to clarify adaptive pattern differentiation. The results showed that northern Chinese isolates had significantly higher survival rates, with cold tolerance closely linked to lysophosphatidylethanolamine (LysoPE). Silencing the LysoPE-related gene BX02G0260 markedly elevated nematode mortality under low temperatures. Unlike native populations, invasive PWN may have developed a cold adaptation strategy centered on genetic material protection, with xanthosine as a key metabolite. These findings provide critical molecular insights into invasive species’ rapid cold adaptation in novel environments.

## 1. Introduction

Cold tolerance is a key adaptive trait enabling invasive alien species (IAS) to successfully establish themselves and expand into temperate and cold regions. Studies have confirmed that compared with sympatric native species, many IAS exhibit stronger cold tolerance [1,2], with core adaptive strategies including maintaining metabolic homeostasis under low-temperature conditions or developing efficient antifreeze mechanisms through genetic evolution and phenotypic plasticity [3,4]. Cold tolerance directly determines whether IAS can break through climatic and geographical barriers and spread to low-temperature areas at high latitudes and altitudes [5]; in-depth analysis of their cold adaptation mechanisms can provide a scientific basis for risk prediction, precise prevention and control of invasive species, and ecosystem protection, while offering important theoretical support for biosecurity governance in the context of global climate change.

*Bursaphelenchus xylophilus* (pine wood nematode, PWN) is one of the most destructive IAS in global forest ecosystems, and its infection can cause rapid wilting and death of pine trees [6,7]. China is one of the countries most severely affected by pine wilt disease (PWD); approximately a quarter of the country’s pine forests, accounting for 25% of the total forest area, are under serious threat from this disease. Since the first detection of PWD in China in 1982, this epidemic has spread to 663 counties across 18 provinces, with an increasingly prominent northward expansion trend [8]. Starting from 2016 onwards, PWD outbreaks have successively occurred in Liaoning and Jilin Provinces in northern China, where the annual average temperature is usually below 10 °C [9,10]. The emergence of these northern epidemic areas has broken the traditional perception that PWD is only distributed south of the isotherm with an annual average temperature of 15 °C [10,11,12]. Nevertheless, this epidemic has still caused severe ecological and economic losses. Whether this expansion into low-temperature regions has led to adaptive evolution has recently become a research hotspot.

Temperature is a key environmental factor regulating the survival and development of PWN [13,14], exerting a significant impact on the nematode’s physiological activities. At the molecular level, existing studies have confirmed that Cytochrome P450 [15], G protein-coupled receptor genes [16], and genes such as Bx-daf-11, Bx-tax-2, and Bx-tax-4 are all involved in cold adaptation [17]. Silencing these genes via RNA interference (RNAi) significantly reduces the cold tolerance of PWN. At the metabolic level, the accumulation levels of trehalose and glycerol in PWN increase significantly under low-temperature stress, and both are considered important cryoprotective substances [18]. The aforementioned studies initially revealed the mechanisms associated with the cold adaptation of PWN. However, as a typical invasive alien species, whether there are differences in cold adaptation between the invasive populations formed during the invasion process and the native populations of PWN, as well as their underlying molecular and metabolic regulatory mechanisms, remains unclear. In this study, an integrated analysis of transcriptomics and metabolomics was employed to systematically investigate the differences in cold adaptation between the native and invasive populations of PWN. We clarified the divergent characteristics of cold adaptation patterns between the native and invasive populations. On this basis, an integrated transcriptome–metabolome analysis was conducted on cold-tolerant PWN strains to mine the key functional genes regulating cold adaptation, and the biological functions of the target genes were verified using RNAi technology. The results of this study provide a new theoretical basis and experimental clues for in-depth analysis of the evolutionary mechanisms underlying the cold adaptation of PWN during its invasion.

## 2. Results

### 2.1. Differences in Cold Tolerance Among PWN Isolates from Different Geographical Origins

**Figure 1:**
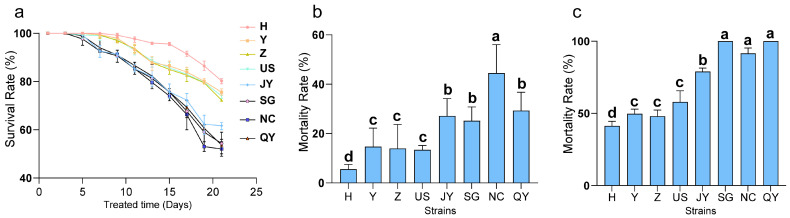
Survival status of eight PWN isolates under low-temperature conditions. (**a**) Survival rate of PWN under continuous treatment at 10 °C for 21 days. (**b**) Mortality rate of PWN after 30 min treatment at −5 °C. (**c**) Mortality rate of PWN after 1 h treatment at −5 °C. Different lowercase letters (a, b, c, d) above the bars indicate significant differences between groups (*p* < 0.05, one-way ANOVA followed by Tukey’s test).

Under the 10 °C treatment, the overall survival rate of the northern Chinese isolates (H, Y, Z) was higher than that of the U.S. isolate (US) and southern Chinese isolates (JY, SG, NC, QY) (Figure 1a). After 30 min of treatment at −5 °C, the mortality rates of the northern Chinese and U.S. isolates were significantly lower than those of the southern Chinese isolates (Figure 1b). Following 1 h of treatment, significant differences in mortality rates were observed among isolates from different regions: northern Chinese isolates showed significantly lower mortality than southern Chinese isolates, and the H isolate exhibited the highest survival rate (Figure 1c). In summary, the cold tolerance test revealed a trend in northern Chinese isolates > U.S. isolate > southern Chinese isolates, with the H isolate demonstrating excellent cold tolerance.

### 2.2. Transcriptomic Responses of Invasive and Native PWN Isolates Under Low-Temperature Stress

**Figure 2:**
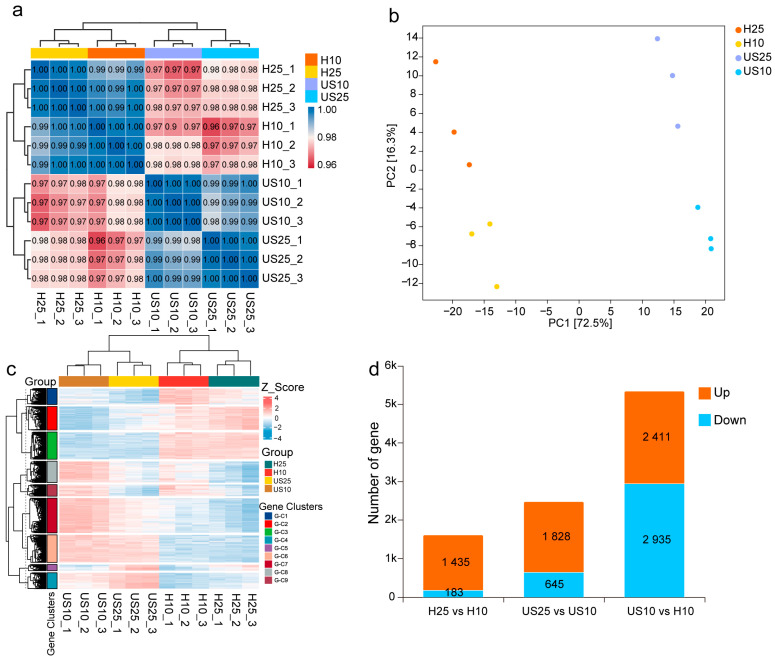
Analysis of gene expression patterns in PWN under low-temperature stress. (**a**) Correlation heatmap—US10_(1–3): native US strain (from America) treated at 10 °C (low temperature); US25_(1–3): native US strain (from America) treated at 25 °C (normal temperature); H10_(1–3): invasive H strain treated at 10 °C (low temperature); H25_(1–3): invasive H strain cultured at 25 °C (normal temperature). (**b**) PCA plot of differentially expressed genes (DEGs). (**c**) Cluster heatmap of DEGs. (**d**) Statistical plot of DEGs: orange boxes indicate upregulation, and blue boxes indicate downregulation. The comparison groups are defined as follows: H25 vs. H10 (the invasive H strain at 25 °C as control vs. the 10 °C treatment group as experimental group, showing DEGs in response to low temperature); US25 vs. US10 (the native US strain at 25 °C as control vs. the 10 °C treatment group as experimental group, showing DEGs in response to low temperature); US10 vs. H10 (the native US strain at the same 10 °C as control vs. invasive H strain as experimental group, showing differences in genes of invasive H strain in response to low temperature compared with native US strain).

The invasive isolate H, with its excellent cold tolerance, and the native isolate US were selected for subsequent RNA-Seq transcriptome validation. As shown in Figure 1a, the *B. xylophilus* strains exhibited a 0% mortality rate (100% survival rate) after 24 h of treatment at 10 °C, with no mortality observed. This indicates that this temperature only induced a cold stress response in the nematodes without causing lethal effects, ensuring that all samples used for transcriptome sequencing were in a viable state and could thus accurately reflect the gene expression changes associated with cold acclimation in the nematodes. Transcriptome sequencing data were generated under two temperatures: 10 °C (H10, US10) and 25 °C (H25, US25). In this scenario, 25 °C served as the normal temperature control and 10 °C as the low-temperature stress treatment. For comparisons among different groups, the R^2^ of all biological replicates was always greater than 0.9 (Figure 2a), indicating a strong correlation between samples. The PCA results showed good consistency of biological replicates within groups (Figure 2b), with significant differences between the low-temperature treatment group and the normal temperature group. Meanwhile, significant differences were also observed between the invasive isolate and the native isolate under the same low-temperature treatment. Cluster heatmaps of each differential group drawn based on FPKM values revealed significant differences in gene expression among different groups (Figure 2c). The above results confirm that the RNA-Seq data obtained in this study have a high reliability and can be used for subsequent analysis of differentially expressed genes (DEGs).

**Figure 3:**
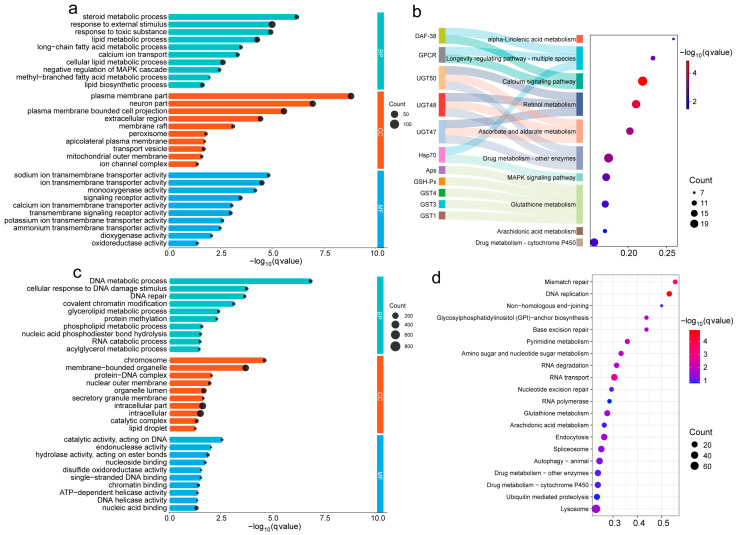
GO and KEGG analyses among different comparison groups. (**a**) GO enrichment analysis of upregulation genes in H25 vs. H10 group, categorized into BP (biological process, green), CC (cellular component, orange), and MF (molecular function, blue). X-axis = −log10(qvalue). (**b**) KEGG pathway enrichment analysis of upregulation genes in H25 vs. H10 group. Left: gene annotations; middle: chord diagram linking genes to pathways; right: bubble plot showing gene ratio and statistical significance (−log10(qvalue)). Bubble size = number of expanded genes per pathway. (**c**) GO enrichment analysis of upregulation genes in US10 vs. H10 group. (**d**) KEGG pathway enrichment analysis of upregulation genes in US10 vs. H10 group.

DEG analysis showed that with 25 °C as the control and 10 °C as the treatment, a total of 1618 DEGs (1435 upregulated; 183 downregulated) were detected in the H25 vs. H10 group; 2473 DEGs (1828 upregulated; 645 downregulated) were identified in the US25 vs. US10 group, under the same low-temperature treatment (10 °C); and 5346 DEGs (2411 upregulated; 2935 downregulated) were screened in the US10 vs H10 group (Figure 2d). GO and KEGG functional enrichment analyses were performed on the aforementioned DEGs. In the H25 vs. H10 comparison group, the GO enrichment results of upregulated DEGs showed that biological processes (BPs) were mainly enriched in the steroid metabolic process, lipid metabolic process, response to external stimulus, and negative regulation of MAPK cascade; molecular functions (MFs) involved calcium ion transmembrane transport activity and ion channel complex; and cellular components (CCs) were concentrated in plasma membrane component, neuron, mitochondrial membrane, and other structures (Figure 3a). The KEGG enrichment results indicated that differential genes were mainly involved in metabolic pathways, such as alpha-linolenic acid metabolism, retinol metabolism, and ascorbic acid metabolism; signal pathways, including calcium signaling pathway, MAPK signaling pathway, and longevity regulating pathway; and defense-related pathways, such as drug metabolism (cytochrome P450-mediated) and glutathione metabolism (Figure 3b). Among them, UGT gene families (UGT47/48/50) were associated with multiple metabolic pathways, suggesting that they play a core role in metabolic regulation under low-temperature stress, and GPCR and DAF-38 gene families were associated with signal pathways and metabolic pathways, respectively, which indirectly confirmed the “signal–metabolism” synergistic response mode during the cold adaptation of PWN. In summary, the cold tolerance of PWN relies on the synergistic effect of multiple pathways: lipid metabolic pathways provide material basis, signal pathways transmit low-temperature stress signals, and defense pathways alleviate cold-induced cellular damage, ultimately enabling adaptation to low-temperature environments. A largely consistent result was also observed during the low-temperature adaptation process in the native range (US25 vs. US10) (Figure S1a,b).

In the US10 vs. H10 comparison group, the GO enrichment results showed that differential genes were significantly enriched in BPs such as the DNA metabolic process, the DNA damage response, and DNA repair, as well as lipid metabolic pathways including glyceride and phospholipid metabolism; MFs were concentrated in DNA binding and nuclease activity (Figure 3c). The KEGG enrichment results were consistent with the GO enrichment results, with significant enrichment of DNA repair-related pathways such as mismatch repair and DNA replication, indicating that the invasive population has enhanced genetic material protection mechanisms under low-temperature conditions. Meanwhile, enrichment of pathways such as pyrimidine metabolism and glucose metabolism can supplement the demand for energy and material synthesis under low-temperature environments; activation of glutathione metabolism, drug metabolism (cytochrome P450-mediated), and other pathways further enhanced the ability to scavenge oxidative damage (Figure 3d). The above results indicate that compared with the native population, the invasive PWN may have evolved a cold adaptation strategy centered on genetic material protection that facilitates its successful survival and colonization in low-temperature regions of northern China.

### 2.3. Differences in Metabolites Related to Low-Temperature Adaptation Between Invasive and Native PWN Isolates

**Figure 4:**
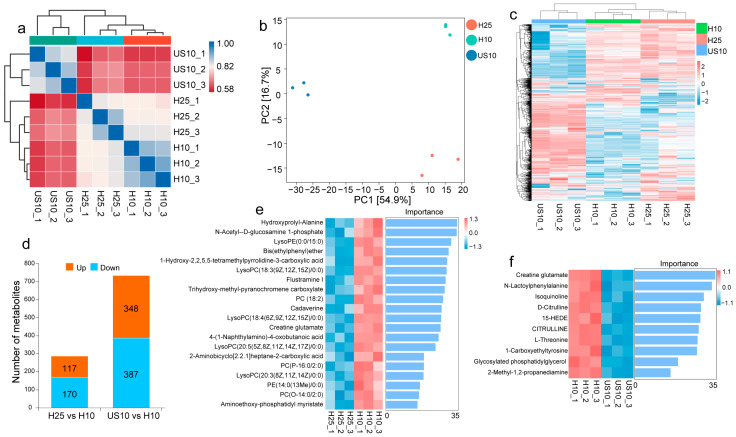
Analysis of metabolite expression patterns in PWN under low-temperature stress. (**a**) Correlation heatmap—US10_(1–3): native US strain (from America) treated at 10 °C (low temperature); H10_(1–3): invasive H strain treated at 10 °C (low temperature); H25_(1–3): invasive H strain cultured at 25 °C (normal temperature). (**b**) PCA plot of differential metabolites (DAMs). (**c**) Cluster heatmap of DAMs, with grouping consistent with Fig. a. (**d**) Statistical plot of DAMs: orange boxes indicate upregulation, and blue boxes indicate downregulation. The comparison groups are defined as follows: H25 vs. H10 (the metabolome of invasive H strain at 25 °C as control vs. the 10 °C treatment group as experimental group, showing DAMs in response to low temperature) and US10 vs. H10 (the metabolome of native US strain at the same 10 °C as control vs. invasive H strain as experimental group, showing differences in metabolites of invasive H strain in response to low temperature compared with native US strain). (**e**) Analysis of the importance of upregulated metabolites in the H25 vs. H10 group. (**f**) Analysis of the importance of upregulated metabolites in the US10 vs. H10 group.

Mass spectrometric analysis of different quality control (QC) samples showed good overlap of total ion current (TIC) chromatograms, with the retention times, peak shapes, and relative intensities of chromatographic peaks in each QC sample exhibiting a highly consistent overall trend and no obvious peak shifts, peak losses, or excessive signal fluctuations, indicating that the metabolite extraction and detection techniques used in this study had reliable repeatability and stability and that the data quality met the requirements for subsequent analyses (Figure S2); a total of 714 metabolites were detected in this study and were classified into 13 categories, among which organic acids and their derivatives were the most diverse (225 species, accounting for 31.5%), followed by lipids and lipid-like molecules (165 species, 23.1%) and organic heterocyclic compounds (115 species, 16.1%), and these three categories collectively accounted for 70.7% of the total detected metabolites (Figure S3), representing the major metabolite classes in the studied system. Sample correlation analysis (Figure 4a), principal component analysis (PCA) (Figure 4b), and differential metabolite clustering results (Figure 4c) all confirmed that the biological replicate samples within each group showed good consistency, while significant differences existed between different temperature treatment groups. For the invasive strain (H) in the introduced range, analysis of differentially accumulated metabolites (DAMs) between the H25 and H10 groups, with 25 °C as the control (H25) and 10 °C as the low-temperature treatment (H10), identified a total of 287 DAMs (including 117 significantly upregulated and 170 significantly downregulated metabolites, with lipids and lipid-like molecules accounting for the highest proportion (32.4%) among the upregulated ones) (Figure 4d; Table S1). For the US10 and H10 groups both exposed to the same 10 °C low-temperature treatment, with the native US strain (US10) from America as the control and the invasive H strain (H10) as the treatment, a total of 735 DAMs were identified (348 upregulated and 387 downregulated). Amino acids, peptides, and analogs constituted 19% of the upregulated metabolites, representing the core category of differential metabolites in this group (Figure 4d; Table S2). Random forest analysis further identified the core signature metabolites responsible for intergroup differences, among which Hydroxyprolyl-Alanine, N-Acetyl-D-glucosamine 1-phosphate, and lysophosphatidylethanolamine (LysoPE) ranked on top in terms of importance among the upregulated metabolites in the H25 vs. H10 group (Figure 4e), while Creatine glutamate and N-Lactoylphenylalanine were the core signature metabolites among the upregulated ones in the US10 vs. H10 group (Figure 4f). These metabolites can effectively distinguish differences between groups and provide key targets for subsequent elucidation of metabolic mechanisms associated with low-temperature adaptation.

### 2.4. Integrated Analysis Reveals Key Genes for Low-Temperature Adaptation

**Figure 5:**
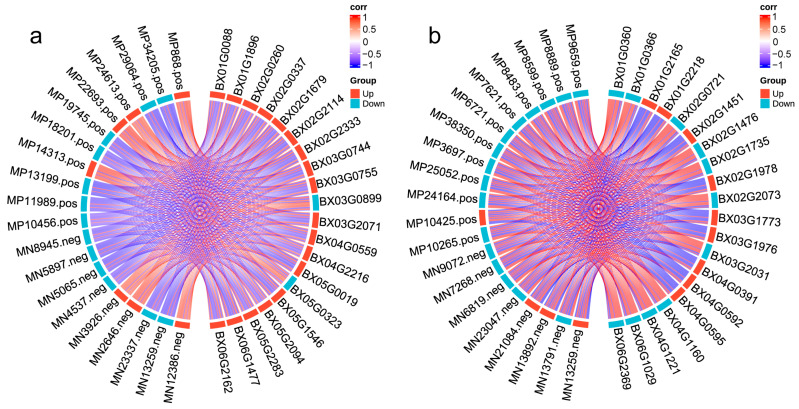
Integrated transcriptomic and metabolomic analysis. (**a**) Integrated transcriptomic and metabolomic analysis of H25 vs. H10. (**b**) Integrated transcriptomic and metabolomic analysis of US10 vs. H10.

We performed an integrated transcriptome and metabolome analysis, and the results revealed a strong positive correlation (R > 0.8) between several genes and metabolites in pairwise group comparisons, indicating that changes in metabolite accumulation may be directly or indirectly regulated by the corresponding genes. We conducted a combined analysis of the H25 vs. H10 and US10 vs. H10 groups. In the H25 vs. H10 comparison, genes BX03G0755, BX03G0744, and BX02G0260 (upregulated expression was all verified by RT-qPCR (Figure S4)) showed a significant positive correlation (R > 0.8) with upregulated metabolites, including LysoPE (MP24613.pos) and β-N-acetylglucosamine (MN12386.neg) (Figure 5a)—previous studies have shown that both metabolites are associated with cold stress responses [19]. This suggests that they may play a critical role in the in situ low-temperature adaptation of the invasive population in the invaded area. Notably, random forest analysis results showed that LysoPE ranked among the top in importance, which echoes the strong gene–metabolite correlation results mentioned above and further confirms the core role of LysoPE in low-temperature adaptation.

In the US10 vs. H10 comparison, the upregulated metabolite xanthosine (MN13892.neg) was well characterized as a functional compound: xanthosine participates in purine metabolism, whereas the hydrolytic product of glucoerucin exhibits direct antioxidant activity; can protect DNA from exogenous compound damage [20,21]; and can enhance the cold tolerance of organisms. The upregulated genes that significantly correlated with xanthosine include HSP90 (BX03G1773), nuclear pore complex protein gene (BX02G1978), and STIP1 (BX03G1976) (Figure 5b). The upregulated expression of these genes was confirmed via RT-qPCR (Figure S4).

### 2.5. RNAi Validation of Low-Temperature-Related Genes

**Figure 6:**
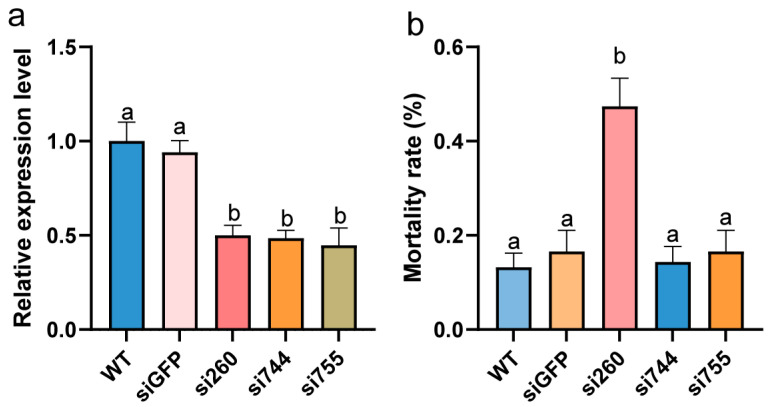
Analysis of silencing efficiency of related genes and mortality under low-temperature stress after silencing. (**a**) Silencing efficiency of related genes. (**b**) Mortality under low-temperature stress after silencing. WT represents untreated nematodes, siGFP serves as the negative control for the silencing operation, si260 denotes nematodes with gene BX02G0260 silenced, si744 refers to nematodes with gene BX03G0744 silenced, and si755 indicates nematodes with gene BX03G0755 silenced. Different lowercase letters (a, b) above the bars indicate significant differences between groups (*p* < 0.05, one-way ANOVA followed by Tukey’s test).

To investigate the key genes involved in the temperature adaptability of PWN in invaded areas, we performed RNAi experiments on three significantly associated genes (BX03G0755, BX03G0744, and BX02G0260) identified from the H25 vs. H10 comparison group, aiming to verify their functions in low-temperature adaptation. The detection results after RNAi showed that the expression levels of siBX03G0755 (si755), siBX03G0744 (si744), and siBX02G0260 (si260) all decreased to below 50% (relative expression level) (Figure 6a). A temperature of −5 °C was the cold-sensitive temperature for *B. xylophilus* (Figure 1b), at which a distinct lethal phenotype was rapidly induced in the nematodes, making this temperature suitable for the quantitative analysis of mortality. After the RNAi-treated nematodes were subjected to low-temperature treatment at −5 °C for 30 min, phenotypic statistical results indicated that there was no significant difference in nematode mortality between the si744 and si755 treatment groups compared with the control group. However, the mortality rate of the si260 treatment group under low temperature was significantly different from that of the control group, reaching 47.4% (Figure 6b). Domain analysis of the BX02G0260 gene revealed that this gene contains an aspartyl protease (ASP) domain. Subcellular localization prediction (http://www.csbio.sjtu.edu.cn/bioinf/euk-multi-2/, accessed on 25 December 2025) indicated that this is localized in the cell membrane. As an essential cellular component, LysoPE’s strong correlation with BX02G0260 led us to hypothesize that the BX02G0260 gene may regulate the fluidity of biological membranes by modulating the content of LysoPE, thereby participating in the low-temperature adaptation process of PWN.

## 3. Discussion

In recent years, PWN has shown a distinct northward expansion trend in China, and its adaptation to low-temperature environments has become a core issue in its invasion into northern China. In this study, an integrated analysis of transcriptomics and metabolomics combined with functional verification via RNAi was performed to systematically investigate the adaptive mechanisms of PWN to the low-temperature environments in invaded areas. The differences in the enriched pathways of DEGs between the two comparison groups reflected the adaptive characteristics of PWN in its invaded regions. Among the DEGs of the nematode population in invaded areas, the pathways were mainly concentrated in membrane lipid remodeling and stress signal transduction. During the dispersal process between the invaded and native populations, the nematodes in invaded areas may have enhanced DNA protection, energy metabolism, glutathione metabolism, and cytochrome P450-mediated drug metabolism, thereby further strengthening their low-temperature adaptation in invaded habitats. The specific enrichment of DNA repair-related pathways also indicated adaptive divergence between the invasive and native populations.

In the integrated analysis of temperature-differential genes and metabolites between the invasive and native ranges, xanthosine exhibited a strong correlation with multiple homeostasis-maintaining genes, such as HSP90 and STIP1 [21,22,23]. Xanthosine is involved in purine metabolism [24], whose pathway not only provides ATP substrates for cellular energy metabolism under low-temperature conditions but also contains intermediate metabolites that participate in the de novo synthesis of nucleotides during DNA damage repair. Meanwhile, it regulates intracellular redox homeostasis and alleviates the damage of reactive oxygen species (ROS) induced by low temperatures to biological macromolecules [20,25]. Purine metabolism involving xanthosine can generate ATP (Cole, 2016) [26], which serves as a key energy molecule for the conformational cycle of HSP90 and its binding to STIP1 [27,28,29], potentially establishing an indirect metabolic link. Compared with native populations, the high correlation between xanthosine and HSP90/STIP1 in invasive populations may represent an adaptive evolutionary outcome shaped by long-term temperature acclimation. By enhancing the synergistic interaction between purine metabolism and the molecular chaperone system, invasive populations have improved their tolerance to the low-temperature environments of invasive ranges. This finding also explains the invasion and expansion capability of this species in high-latitude, low-temperature regions. This correlation provides key molecular evidence for the rapid adaptation of invasive species to low-temperature stress in novel environments.

The metabolomic results demonstrated that LysoPE acts as a core signature metabolite and is one of the most critical regulators for low-temperature adaptation. Membrane fluidity is one of the key factors for organisms to cope with low-temperature stress. Low temperatures tend to induce membrane lipid rigidification, which impairs the activities of membrane-bound enzymes, transmembrane transport, and cellular signal transduction, among other physiological processes [30,31]. Lipid metabolism plays a critical role in the cold acclimation of most species [32,33]. During the low-temperature storage of bitter gourds, lysophosphatidylcholine (LysoPC) and LysoPE, two key phospholipid metabolites, have been reported to be involved in cold resistance responses [34]. LysoPE can maintain membrane fluidity by altering membrane lipid composition and phase behavior, which may facilitate cellular adaptation to low-temperature stress. As an important membrane lipid component [35], LysoPE is likely to play a key role in the low-temperature adaptation mechanism of PWN. Further verification via RNAi demonstrated that silencing the associated gene BX02G0260 (aspartic protease) significantly increased the mortality rate of nematodes under low-temperature conditions. In fungi, phosphatidylethanolamine (PE) enhances the proteolytic activity of the aspartic protease derived from Mucor miehei [36]; in addition, studies on an integral membrane aspartic protease from the halophilic archaeon H. volcanii have indicated that its oligomerization state is lipid-dependent [37]. As a derivative of PE, LysoPE is interconvertible with PE via cellular metabolic pathways, jointly maintaining lipid homeostasis [38]. All these findings confirm that aspartic proteases and lipid metabolism are intricately correlated. This study is the first to confirm the antifreeze function of LysoPE in invasive nematodes, supplementing the lipid regulatory mechanism underlying the low-temperature adaptation of PWN.

Although the core regulatory pathways, key genes, and metabolites have been identified, the regulatory mechanisms between genes and metabolites remain unclear. Additional molecular experiments are required in future studies to elucidate these mechanisms. Nevertheless, this study clarified the preliminary mechanism of low-temperature adaptation in PWN, identified the core antifreeze metabolite LysoPE and the key functional gene BX02G0260, and also preliminarily explored the metabolic pathways and differential metabolites of nematodes from invasive and native ranges at low temperatures, thereby providing a new molecular basis for understanding the rapid adaptive evolution of invasive species.

## 4. Materials and Methods

### 4.1. Sample Collection

Diseased pine wood samples were collected from epidemic areas across three provinces of China (Table 1). Nematodes in the infected wood were isolated via the Baermann funnel technique, followed by morphological identification under a microscope [8]. Molecular biological identification was then performed using specific primers [39]. In total, eight isolates of PWN were obtained (Table 1). All the isolated PWN strains were inoculated onto a Botrytis cinerea culture medium and incubated at 25 °C to obtain sufficient samples for subsequent experiments [40]. The nematode isolates were then preserved in sterile water using 1.5 mL centrifuge tubes.

### 4.2. Cold Treatment and Survival Rate Counting

Nematode suspensions (200 individuals per replicate) were transferred into 1.5 mL centrifuge tubes containing 1 mL of sterile distilled water. For the 10 °C mortality assessment, the experiment was performed with 3 biological replicates, and tubes were incubated at 10 °C. Over a 21 day experimental period, subsamples of nematodes from each group were transferred to 24 well microplates at 3 day intervals. Nematode viability was evaluated via mechanical stimulation under a microscope: individuals were gently prodded with a glass capillary tube; those exhibiting overt whole-body curling or subtle head/tail movements were categorized as viable, while immobile individuals were scored as non-viable. Mortality rate was calculated for each technical replicate, and the mean values from the 3 technical replicates were used to represent the result of each biological replicate. For the mortality assay at −5 °C, the same experimental design with 3 biological replicates was used, and the centrifuge tubes were subjected to low-temperature treatment in a −5 °C cryobath for 30 min and 1 h, respectively. Subsequently, the tubes were transferred to a 25 °C incubator for a 24 h recovery period. The mortality rate of the nematodes was determined using the identical method described above.

### 4.3. Transcriptome Sequencing and Differential Expression Analysis

The cold-tolerant strain H isolated from the invasive range and the US strain from the native range were separately cultured at 25 °C and 10 °C for 24 h for RNA sequencing analysis. Total RNA was isolated using the TRIzol^®^ reagent (Invitrogen, Carlsbad, CA, USA). RNA concentration and integrity were assessed with a NanoDrop ND-1000 spectrophotometer (Thermo Fisher Scientific, Waltham, MA, USA). RNA sequencing was performed on the Illumina NovaSeq™ 6000 platform (Illumina, San Diego, CA, USA) with the paired-end 150 bp (PE150) strategy by Personal Biotechnology Co., Ltd. (Shanghai, China). Raw reads were quality-filtered using fastp v1.0.1 [41], and clean reads were aligned to the reference genome (TS-1) via HISAT2 v2.2.1 [42,43]. Transcript assembly and expression quantification (FPKM values) were conducted using StringTie v3.0.1 [44]. Differentially expressed genes (DEGs) were identified with the DESeq2 package in R v4.5.2 [45], using the screening thresholds of fold change ≥ 1.2, raw *p* ≤ 0.05, and false discovery rate (FDR, i.e., q-value) ≤ 0.05 after Benjamani–Hochberg correction for multiple testing. Functional annotation of DEGs was carried out via GO and KEGG enrichment analyses [46,47], with visualization implemented in R v4.5.2.

### 4.4. Non-Targeted Metabolomics Analysis

Thirty milligrams of each sample was accurately weighed into a 2 mL centrifuge tube, and a methanol/acetonitrile solution (1:1, *v*/*v*) was added thereto. The mixture was vortexed for 30 s and then homogenized for 30 min, followed by incubation at −20 °C for 10 min. After centrifugation at 18,400× *g* and 4 °C for 20 min, 200 μL of the supernatant was collected to prepare samples for LC-MS analysis [48]. Quality control (QC) samples were prepared by pooling equal volumes of extracts from all samples, with the volume of the QC samples consistent with that of the test samples. LC-MS analysis was performed on a high-resolution LC-MS platform from Thermo. Chromatographic conditions: ACQUITY UPLC HSS T3 column (100 Å, 1.8 μm, 2.1 mm × 100 mm), where the mobile phases consisted of mobile phase A (0.1% formic acid in water, *v*/*v*) and mobile phase B (0.1% formic acid in acetonitrile, *v*/*v*). The gradient elution program was set as follows, with a total analysis cycle of 8.5 min: 0–1.0 min, mobile phase B was maintained at 5%; 1.0–4.7 min, mobile phase B was linearly increased to 95%; 4.7–6.0 min, mobile phase B was maintained at 95%; 6.0–6.1 min, mobile phase B was rapidly decreased to 5%; and 6.1–8.5 min, mobile phase B was maintained at 5% to equilibrate the column. The flow rate was 0.4 mL/min, column temperature was 40 °C, and injection volume was 2 μL. Mass spectrometric conditions: Thermo Orbitrap Exploris 120 mass spectrometer controlled by Xcaliburv4.3, with data-dependent acquisition (DDA) in positive/negative ion modes. HESI source parameters: spray voltage 3.5 kV/−3.0 kV, capillary temperature 320 °C; MS1 resolution 60,000 (scan range 70–1000 *m*/*z*), MS2 resolution 15,000 (HCD collision energy 30%). Raw data were processed using MS-DIAL v4.9 for peak extraction, alignment, and filtering, as well as metabolite annotation. Missing values were imputed via the Gap filling algorithm, followed by data normalization. Metabolite identification was achieved by matching the accurate mass and MS2 spectra against the PSNGM database. The principal component analysis (PCA) and orthogonal partial least squares–discriminant analysis (OPLS-DA) were performed using SIMCA v16.0 on all identified metabolites to investigate global metabolic variations, intergroup differences, and intragroup sample discrepancies. Based on the OPLS-DA results, metabolites with variable importance in projection (VIP) ≥ 1, fold change ≥ 1.2, raw *p* ≤ 0.05, and q-value ≤ 0.05 (calculated via the Benjamani–Hochberg method for multiple testing correction) were selected as differential metabolites. To further assess the differences between the metabolites, the random forest model was implemented using R v4.5.2 (randomForest package, version 4.7-1.1) for category prediction based on majority voting from the ensemble [49].

### 4.5. Combined Transcriptomics and Metabolomics Analyses

Differentially accumulated metabolites (DAMs) and differentially expressed genes (DEGs) were analyzed using MetaboAnalyst v5.0, which was applied to simultaneously map the DAMs and DEGs onto KEGG pathway diagrams by integrating metabolomic and transcriptomic data. Correlation analysis was performed on the top 20 genes and metabolites detected in each sample group. Pearson’s correlation coefficients between genes and metabolites were calculated using the COR function in R software. The correlation coefficients between metabolites and genes were visualized as network graphs, with Pearson’s correlation coefficient > 0.8 for each group.

### 4.6. In Vitro RNAi

Gene-targeting dsRNAs and GFP dsRNA were synthesized using the Vazyme T7 in vitro RNAi Transcription Kit (TR102, Vazyme Biotech Co., Ltd., Nanjing, China). Mixed-stage nematodes were incubated with 500 ng/μL of each dsRNA (or dsRNA-free control) at 25 °C with shaking (100 rpm) for 12 h. Silencing efficiency was validated via RT-qPCR, with primers listed in Table 2. All assays were conducted in triplicate [50].

### 4.7. Real-Time Quantitative Polymerase Chain Reaction (RT-qPCR) Detection

cDNA was synthesized from 2 μg total RNA using a HiScript II 1st Strand cDNA Synthesis Kit (+gDNA wiper, Vazyme Biotech Co., Ltd., Nanjing, China). RT-qPCR was performed with PerfectStart Green qPCR SuperMix (TransGen Biotech Biotech Co., Ltd., Beijing, China) on a Roche LightCycler 96 real-time PCR system (Roche Diagnostics, Basel, Switzerland). The actin gene (primers: actin-F/actin-R) was used as the endogenous control [51]. Non-specific amplification was monitored via melting curve analysis, and relative gene expression was calculated via the 2^−ΔΔCt^ method. Primer sequences are listed in Supplemental Table S3.

### 4.8. Assays for Nematode Sensitivity to Low Temperature After Gene Silencing

To evaluate changes in low-temperature sensitivity following gene silencing, the silenced nematodes were soaked in sterile water, exposed to −5 °C for 30 min, and then incubated at 25 °C for 24 h for recovery. Nematode mortality was calculated using the same method described previously.

## Figures and Tables

**Table 1:** List of eight PWN strains collected from different areas.

ID	Sample Origin	ID	Sample Origin
US	The United States	QY	Qingyuan City, Guangdong Province, China
JY	Jiyuan City, Guangdong Province, China	SG	Shaoguan City, Guangdong Province, China
NC	Nanchang City, Jiangxi Province, China	H	Fushun City, Liaoning Province, China
Y	Fushun City, Liaoning Province, China	Z	Fushun City, Liaoning Province, China

**Table 2:** Primers required for gene silencing.

Primer Name	Sequence (5′-3′)
GFP-siRNA-right primer	TAATACGACTCACTATAGGGAGAATGAGTAAAGGAGAAG
GFP-siRNA-left primer	TAATACGACTCACTATAGGGAGAT TTGTATAGTTCATCCATG
RNAi-T7BX03G0755-f	TAATACGACTCACTATAGGGAAGCTGGGCGATCAAGAGTC
RNAi-T7BX03G0755-r	TAATACGACTCACTATAGGGATGCTTAATTCGGCTGAATA
RNAi-T7BX02G0260-f	TAATACGACTCACTATAGGGATGGGCATCATCAAGTCGTT
RNAi-T7BX02G0260-r	TAATACGACTCACTATAGGGCGTAATCAGCGGAGTTGTCA
RNAi-T7BX03G0744-f	TAATACGACTCACTATAGGGAGAGATATACCAGGACAAGG
RNAi-T7BX03G0744-r	TAATACGACTCACTATAGGGAATCTTTCCATCCGTTCGTC

## Data Availability

The original contributions presented in this study are included in the article/Supplementary Material. Further inquiries can be directed to the corresponding authors.

## Supplementary Materials

The following supporting information can be downloaded at https://www.mdpi.com/article/10.3390/ijms27031470/s1.
