# Supplementary material for: Combined Transcriptomic and Metabolomic Analyses of Low-Temperature Adaptation in Bursaphelenchus xylophilus
Source: Int J Mol Sci. 2026 Feb 2;27(3):1470. doi: 10.3390/ijms27031470 (PMC12897806; doi:10.3390/ijms27031470)
Supplement: Supplementary file 1 [file ijms-27-01470-s001.zip › Supplementaty Figures.pdf]

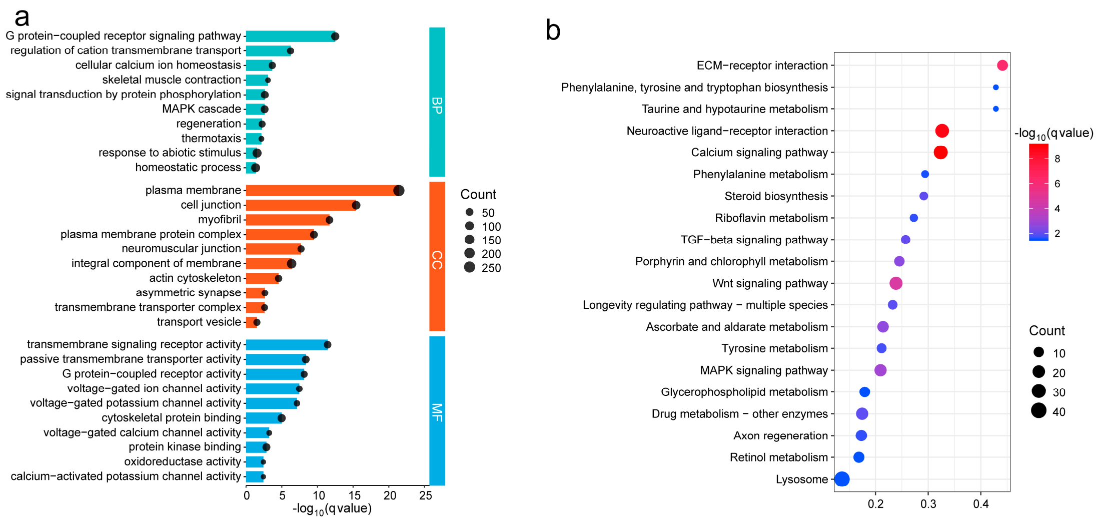

Figure S1. GO and KEGG analyses US25 vs US10 group (a) GO enrichment analysis of up-regulation genes. (b) KEGG pathway enrichment analysis of up-regulation genes.

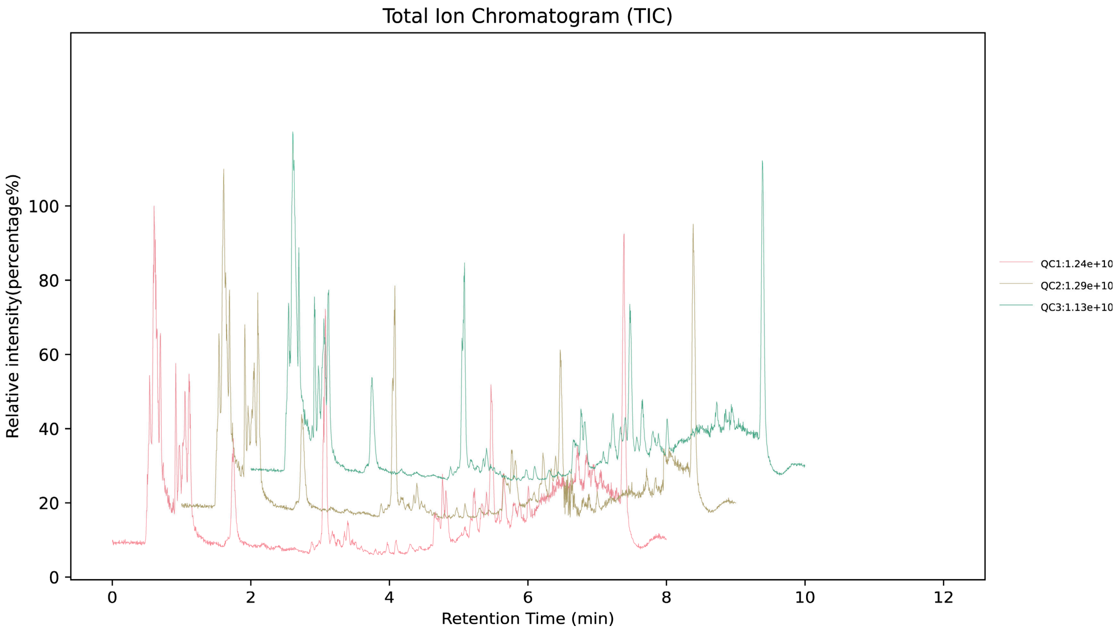

Figure S2. TIC overlay plot for mass spectrometry detection of QC samples

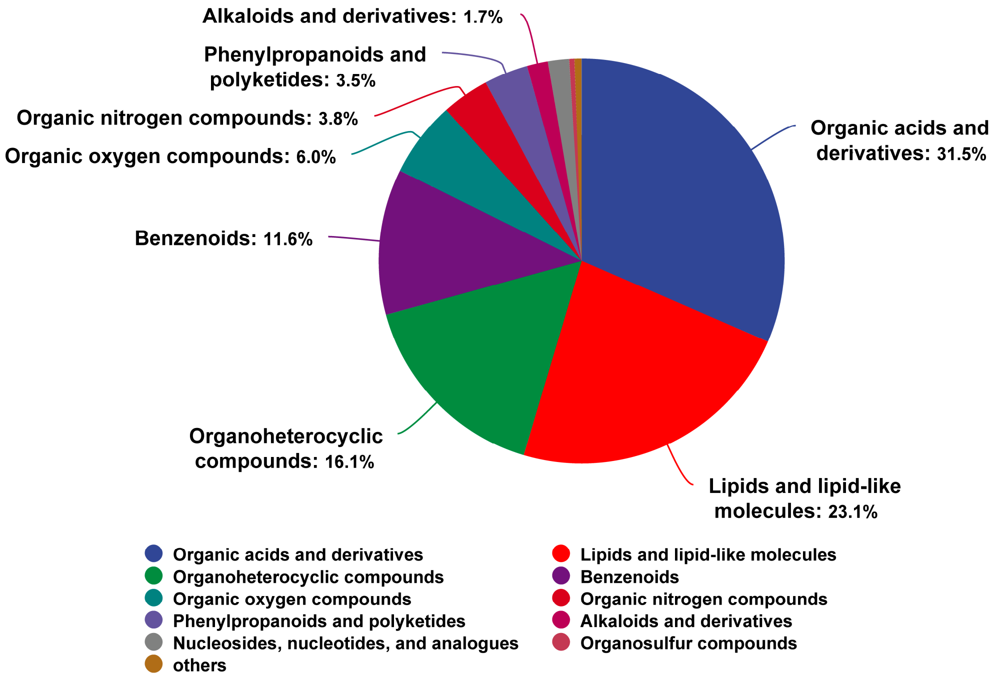

Figure S3. Pie chart of various differential metabolite contents

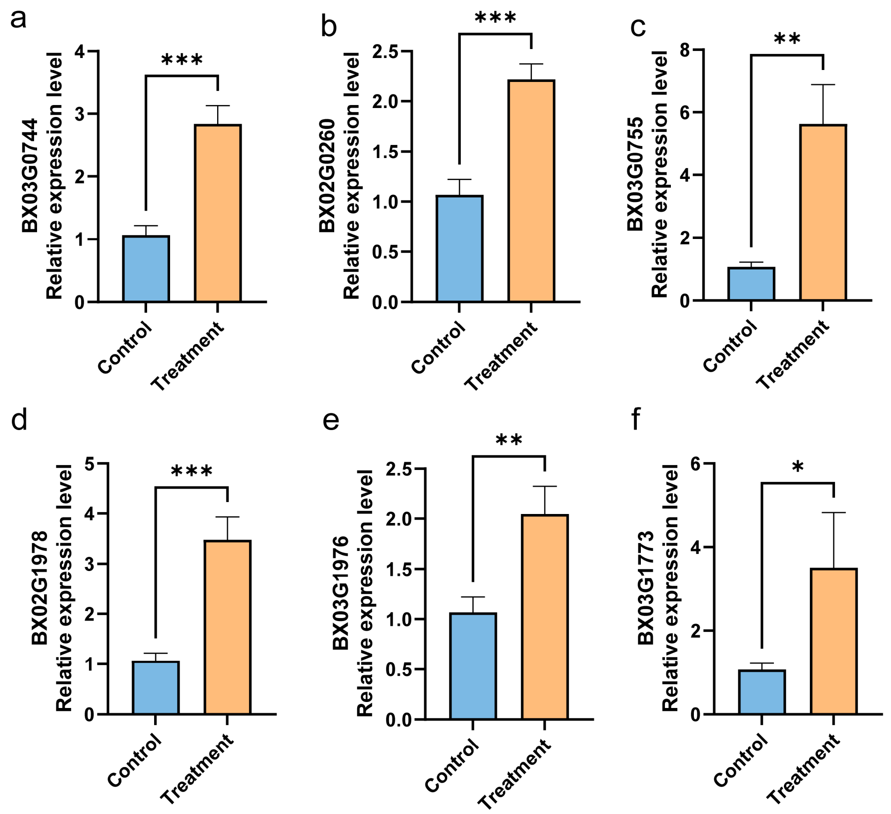

Figure S4. The expression levels of related genes were validated by RT-qPCR.
